# Human Bocavirus in Hospitalized Children, South Africa

**DOI:** 10.3201/eid1209.051616

**Published:** 2006-09

**Authors:** Heidi Smuts, Di Hardie

**Affiliations:** * University of Cape Town, Cape Town, South Africa

**Keywords:** Human bocavirus, respiratory tract infection, South Africa, letter

**To the Editor:** In recent years, several novel respiratory viruses have been identified. These include human metapneumovirus (HMPV) (*1*), severe acute respiratory syndrome–associated coronavirus (*2*), human coronavirus (HCoV) NL63 (*3**,**4*), HCoV HKU1 (*5*), and, most recently, human bocavirus (HBoV) (*6*). The latter belongs to the *Parvoviridae* family and is most closely related to bovine parvovirus and canine minute virus (CnMV), which are members of the genus *Bocavirus* (*6*). Parvovirus B19 and HBoV are the only 2 parvoviruses known to be pathogenic to humans, but the relevance of HBoV infection in the clinical setting is not known.

In this retrospective study, 341 nasopharyngeal and bronchoalveolar lavage samples were taken from children (age 2 days–12 years) hospitalized with respiratory tract infections in 2004 in the Red Cross War Memorial Children's Hospital, Cape Town, South Africa. Samples were originally screened by using an indirect immunofluorescence assay (Light Diagnostics, Chemicon International, Temecula, CA, USA) for common respiratory viruses, including respiratory syncytial virus; influenza virus A and B; parainfluenza viruses 1, 2, and 3; adenovirus; and cytomegalovirus. Subsequently, HMPV and HCoV NL63 were detected by using reverse transcription–PCR (*1**,**3*).

Samples were also screened for HBoV DNA. DNA was extracted by using the QIAamp DNA blood mini kit according to the manufacturer's instructions (Qiagen Inc., Valencia, CA, USA). PCR amplification of a region of the NP-1 gene and the 3´ portion of the VP1/2 capsid gene of HBoV was performed. Briefly, 10 μL DNA was added to a 50-μL PCR mix containing 2 IU Supertherm polymerase (JMR Holdings, Kent, UK), 1.5 mmol/L MgCl_2_, 200 μmol/L each dNTP, and 0.2 μmol/L primers NP-1 s1 (5´-TAACTGCTCCAGCAAGTCCTCCA) and NP-1 as1 (5´-GGAAGCTCTGTGTTGACTGAAT). To improve sensitivity, a second seminested reaction with 2.5 μL outer product and NP-1 as1 primer and NP-1 s2 (5´-CTCACCTGCGAGCTCTGTAAGTA) primer was performed at an annealing temperature of 55°C. Negative controls were used, and appropriate measures were taken to prevent contamination (*7*). Samples with an NP-1–specific PCR product of 368 bp were confirmed by amplifying a 980-bp product of the VP1/2 capsid gene in a similar seminested PCR amplification protocol (primers VP s1 5´-GCACTTCTGTATCAGATGCCTT, VP as1 5´-CGTGGTATGTAGGCGTGTAG, and VP s2 5´-CTTAGAACTGGTGAGAGCACTG). A selection of the inner VP1/2 amplicons obtained from samples taken over the year were sequenced directly and aligned in ClustalX, and a phylogenetic tree was constructed with the Kimura 2-parameter neighbor-joining method with 1,000 bootstrap resamplings. Comparative sequences were obtained from GenBank and included HBoV isolate st1 (DQ000495), HBoV isolate st2 (DQ000496), and a CnMV isolate (NC_004442). Nucleotide sequences from this study were deposited into GenBank (DQ317539–DQ317561).

HBoV DNA was detected in 38 (11%) samples from 35 children, all <2 years of age. Infections occurred throughout the year, although more positive results were found in the autumn/winter season from April to August (63%) than during the rest of the year (37%). A diagnosis of pneumonia or lower respiratory tract infection was made for 30 (86%) children. Thirteen (37%) HBoV-positive children required admission to the intensive care unit. Comorbid conditions were present in 22 children: cystic fibrosis (1), spinal muscular atrophy type 1 (4), Down syndrome (4), cardiac abnormalities (5), and HIV infection (8). Co-infection with a range of viral and bacterial organisms was a common feature in HBoV-positive children and was found in 14 (37%) samples. These organisms included cytomegalovirus (4), respiratory syncytial virus (2), adenovirus (1), HCoV NL63 (1), parainfluenza 3 (1), *Staphylococcus aureus* (1), *Streptococcus pneumoniae* (1), *Klebsiella pneumoniae* (1), and *Pneumocystis jirovecii* (2). However, in the remaining 24 (63%) samples, no other infectious agent was identified.

HBoV was detected in serial samples from 2 children during a 2-day (V04/2591 and V04/2613) and 7-day (V04/2599 and V04/2631) period. In both, sequences were identical and clustered within the proposed subgroup B. In a third child, HBoV sequences were detected in 2 samples taken 2 months apart; in these samples, the isolates were different (V04/1159 and V04/2062) (Figure).

**Figure:**
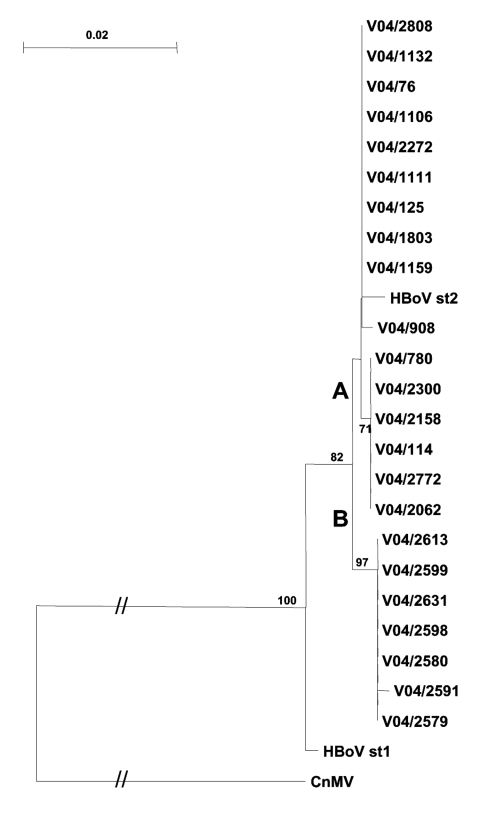
Phylogenetic analysis of a 980-bp region of the human bocavirus (HBoV) VP1/2 capsid gene from South African children with respiratory tract disease. The tree was constructed by using the neighbor-joining method with 1,000 bootstrap resamplings. All nucleotide sequences were submitted to GenBank (accession nos. DQ317539–DQ317561). CnMV, canine minute virus.

Phylogenetic analysis of the 3´ region of the VP1/2 capsid gene (Figure) showed that the Cape Town strains of HBoV were most closely aligned with the HBoV st2 prototype strain. The nucleotide sequence homology was 98% with 1 amino acid change, N474S. The HBoV st2 branch could be separated into 2 lineages, A and B, with a 3-nucleotide change at positions 4615 (A/G), 4756 (A/C), and 4888 (G/A) on the basis of the numbering of the HBoV st2 sequence.

These results suggest that HBoV infection occurs predominantly during the winter season and that children <2 years of age are most at risk. The study by Sloots et al. (*8*) also found HBoV infections mainly during the winter months (61%) in children <2 years. Although co-infections were found, the proportion (63%) of children in whom only HBoV was detected was substantial. These findings suggest that HBoV may play a role in respiratory tract infections in young children who require hospitalization.
